# Effects of different principles of Traditional Chinese Medicine treatment on TLR7/NF-κB signaling pathway in influenza virus infected mice

**DOI:** 10.1186/s13020-018-0199-4

**Published:** 2018-08-20

**Authors:** Ying-Jie Fu, Yu-Qi Yan, Hong-Qiong Qin, Sha Wu, Shan-Shan Shi, Xiao Zheng, Peng-Cheng Wang, Xiao-Yin Chen, Xiao-Long Tang, Zhen-You Jiang

**Affiliations:** 1 Department of Microbiology and Immunology, School of Basic Medical Sciences, Jinan University, Guangzhou, 510632 Guangdong China; 2 College of Traditional Chinese Medicine, Jinan University, Guangzhou, 510632 Guangdong China; 3 Medical College, Anhui University of Science & Technology, Huainan, 232001 Anhui China

**Keywords:** Influenza virus, Traditional Chinese Medicine, TLR7/NF-κB signaling pathway, TLR7 gene knockout, Guizhi-and-Mahuang decoction, Yinqiao powder, Xinjiaxiangruyin

## Abstract

**Background:**

Influenza virus is a single-stranded RNA virus that causes influenza in humans and animals. About 600 million people around the world suffer from influenza every year. Upon recognizing viral RNA molecules, TLR7 (Toll-like receptor) initiates corresponding immune responses. Traditional Chinese Medicines (TCMs), including Yinqiao powder, Xinjiaxiangruyin and Guizhi-and-Mahuang decoction, have been extensively applied in clinical treatment of influenza. Although the therapeutic efficacy of TCMs against influenza virus in vivo was reported previously, its underlying mechanisms are not clearly understood. This study aimed to investigate the immunological mechanisms in the treatment of influenza virus infected mice with three Chinese herbal compounds as well as the effect on TLR7/NF-κB signaling pathway during recovery.

**Methods:**

Wild type and TLR7 KO C57BL/6 mice were infected with influenza virus FM1 and then treated with three TCMs. The physical parameters of mice (body weight and lung index) and the expression levels of components in TLR7/NF-κB signaling pathway were evaluated.

**Results:**

After viral infection, Guizhi-and-Mahuang decoction and Yinqiao powder showed better anti-viral effect under normal condition. Compared to the viral control group, expression levels of TLR7, MyD88, IRAK4 and NF-κB were significantly reduced in all treatment groups. Furthermore, the three TCM treatment groups showed poor therapeutic efficacy and no difference in viral load compared to the viral control group in TLR7 KO mice.

**Conclusion:**

Our study indicated that Guizhi-and-Mahuang decoction and Yinqiao powder might play a crucial role of anti-influenza virus by regulating TLR7/NF-κB signal pathway.

**Electronic supplementary material:**

The online version of this article (10.1186/s13020-018-0199-4) contains supplementary material, which is available to authorized users.

## Background

Influenza virus causes seasonal epidemics and occasional pandemics in human beings and presents serious public health and economic problems [1]. Influenza viruses belong to the *Orthomyxoviridae* family, and are classified into three types, A, B, and C, in which type A virus (influenza A virus) is a major zoonotic pathogen [2]. Influenza A (H1N1) causes an acute respiratory infectious disease with symptoms including fever, cough, diarrhea or vomiting, muscle pain or fatigue, redness of eyes and even death [3]. The most effective means of protection against influenza is vaccination, but its effectiveness has been limited because etiological influenza A and B viruses constantly undergo antigenetic changes [4]. Several anti-influenza drugs have been developed, including M2 protein inhibitors and neuraminidase inhibitors (NAIs). However, nearly all influenza A (H3N2) viruses and part of influenza A (H1N1) viruses are adamantane resistant nowadays, which leaves NAIs the only option for the infection with these viruses [5]. Two NAIs, oseltamivir and zanamivir, are FDA approved for use against type A and type B influenza infections [6]. However, during the 2007–08 influenza season, emergence and transmission of oseltamivir-resistant type A (H1N1) viruses, with an H274Y mutation in the neuraminidase, were observed in several countries in the Northern Hemisphere and then spread globally [7]. In recent years, researchers have also identified oseltamivir resistant influenza type A (H5N1) and type B viruses [5], which makes it urgent for the research and development of new and effective anti-influenza drugs.

Traditional Chinese Medicine (TCM) is an important means to prevent and control influenza in China. Yinqiao powder is a classic prescription from “Wen Bing Tiao Bian”, a TCM compound created by Jutong Wu in Qin Dynasty. As a representative of cool acrid exterior-resolving method of TCM, Yinqiao powder is effective in preventing and treating viral infection diseases, which belong to the category of TCM warm diseases, for its antipyretic and anti-inflammatory effect. Yinqiao powder is commonly used for the prevention of influenza in clinic, and has been widely used in the treatment of influenza A [8]. As a recommended drug against wind-heat offender, heat-infected lung influenza, it was included in the latest Influenza Diagnosis and Treatment Program (2018 edition) by the Chinese National Health and Family Planning Commission. Researches have confirmed that Yinqiao powder contains chlorogenic acid [9], phillyrin [10, 11] and arctiin [12, 13], which showed pharmacological effects of anti-influenza virus.

Xinjiaxiangruyin is also a TCM compound from “Wen Bing Tiao Bian” to prevent dampness and heat, and is mainly used for the treatment of summer fever and heat stroke etc. Some scholars used Xinjiaxiangruyin to treat influenza virus infected mice in hot and humid environment, and found that it had remarkable anti-viral effect [14]. Although it consists of five herbs, Xinjiaxiangruyin has a precise combination, in which elsholtzia mainly contains flavonoids and coumarin compounds, which show heat-clearing dampness, anti-bacterial, anti-inflammatory and other pharmacological effects [15]. In addition, magnolol, the main component of Cortex Magnoliae Officinalis, has antioxidative [16, 17], inflammation regulation [18], cancer cell inhibition [19] and other effects. At the same time, honokiol also has an anti-viral activity [20, 21].

Guizhi-and-Mahuang decoction is from classic Chinese medicine works “typhoid fever” written by Mr. Zhang Zhongjing and has acrid-warm herbs for relieving superficies and sweating slightly. Modern pharmacological studies suggest that Guizhi-and-Mahuang decoction has the functions of anti-influenza virus [22], antipyretic analgesia [23], and anti-inflammatory and asthma [24]. Ephedrine has been used in clinic in the past hundreds of years since its discovery. The studies show that ephedrine plays a role in many organs and tissues, and its mechanism is complex, which involves various types of adrenergic receptors [25]. Cinnamon aldehyde contained in Ramulus Cinnamomi can inhibit the infection of influenza A/PR/8 virus [26] and viral myocarditis [27]. However, although these three Chinese herbal compounds can be used for anti-influenza therapy, the molecular signaling pathway involved remains to be clarified.

It is well established that Toll-like receptors (TLRs) are a major family of pattern recognition receptors [28] and play a crucial role in the recognition of microbial pathogens, thereby inducing innate immune responses in mammalian hosts [29]. Toll-like receptor 7 (TLR7) is expressed within intracellular vesicles [30] and recognizes the single-stranded RNA viruses, like vesicular stomatitis virus and influenza virus [31, 32]. It’s highly expressed by plasmacytoid dendritic cells (DCs) and B cells [32], and involves in the pathways used by the innate immune cells in the recognition of viral pathogens [31]. The TLRs signaling can be divided into two signal transduction pathways [33]. First, myeloid differentiation primary response 88 (MyD88) associates with TLRs through TIR (Toll/IL1-receptor homologous region) to form a complex that recruits the downstream signal molecule interleukin-1 receptor-associated kinase 4 (IRAK4). Phosphorylation of IRAK4 activates interleukin-1 receptor-associated kinase 1 (IRAK1), which subsequently promotes the activation of TNF receptor associated factor 6 (TRAF6). Activated TRAF6 binds to ubiquitin conjugating enzyme (E2) to degrade IKK-gamma and activate TGF-beta activated kinase 1 (TAK1). Activated TAK1 catalyzes the phosphorylation of IKK- beta protein and forms a complex. The phosphorylation results in translocation of nuclear factor kappa-light-chain-enhancer of activated B cells (NF-κB) related gene from cytoplasm into nucleus and activates the downstream mitogen-activated protein kinases (MAPK) pathway, thereby inducing the formation of activator protein-1 (AP-1), and production of inflammatory cytokines such as interleukin-6 (IL-6), interleukin-12 (IL-12) and Tumor Necrosis Factor-α (TNF-α) [34]. Since NF-κB is activated by a large number of stimuli, tight molecular feedback loops normally prevent sustained cellular responses and excessive inflammation. During infection, some influenza viral particles are degraded by endosomal proteases, releasing the viral genome RNA and initiating TLR7 signaling [35, 36].

In this study, we first confirmed that influenza virus infection indeed activated the TLR7/NF-κB signaling pathway. We then infected the wild type and TLR7 KO mice with influenza virus, and applied three TCMs to evaluate their effects on lung injury recovery. Furthermore, expression levels of components in TLR7/NF-κB pathway were detected and the possible immunological mechanisms were explored.

## Methods

### Information of experimental design and resources

The information regarding the experimental design, statistics, and resources used in this study are attached in the minimum standards of reporting checklist (Additional file 1).

### Drug preparation and HPLC establishment

**Fig. 1:**
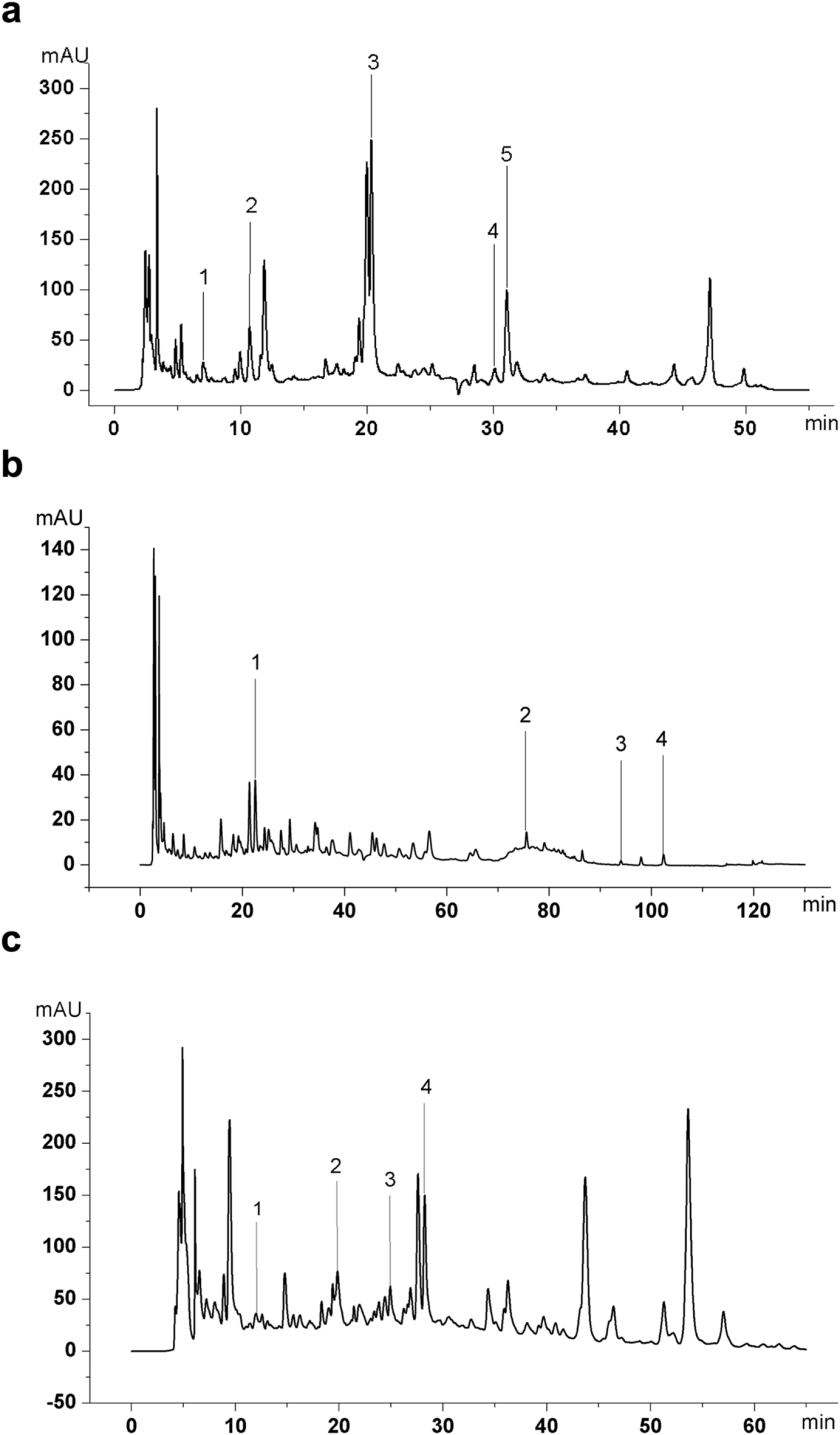
The fingerprints of TCMs. **a** The fingerprint of Yinqiao powder; peak number and identity, 1: 3,4-dihydroxybenzoic acid; 2: chlorogenic acid; 3: liquiritin; 4: forsythin; 5: arctiin. **b** The fingerprint of Xinjiaxiangruyin; peak number and identity, 1: chlorogenic acid; 2: forsythin; 3: thymol; 4: magnolol. **c** The fingerprint of Guizhi-and-Mahuang decoction; peak number and identity, 1: ephedrine hydrochloride; 2: amygdalin; 3: paeoniflorin; 4: liquiritin

Yinqiao powder (15 g Fructus Forsythiae, 15 g Flos Lonicerae, 9 g Radix Platycodonis, 9 g Herba Menthae, 6 g Herba Lophatheri, 5 g Radix Glycyrrhizae, 6 g Herba Schizonepetae, 6 g Fermented soybean, 6 g Fructus arctii, 10 g Rhizoma Phragmitis); Xinjiaxiangruyin (6 g Herba Moslae, 9 g Flos Lonicerae, 9 g Dolichos, 6 g Cortex Magnoliae Officinalis, 6 g Fructus Forsythiae). Guizhi-and-Mahuang decoction contained equal part of Mahuang Tang (9 g Herba Ephedrae, 6 g Ramulus Cinnamomi, 9 g Semen Armeniacae Amarum, 6 g Radix glycyrrhizae preparata) and Guizhi Tang (9 g Ramulus Cinnamomi, 9 g Radix Paeoniae Alba, 6 g Radix Glycyrrhizae, 9 g Rhizoma Zingiberis Recens, 3 g Jujube), which were dissolved in water and combined; These three drugs were all Chinese patented granules, which were purchased from China Resources Sanjiu Medical & Pharmaceutical Co., Ltd. (Table 1). Oseltamivir Phosphate Capsules was obtained from Yichang Yangtze River East Sunshine pharmaceutical Ltd (Lot H20065415). 3,4-dihydroxybenzoic acid (Lot 170707); chlorogenic acid (Lot 171110); liquiritin (Lot 171222); forsythin (Lot 171103); arctiin (Lot 180207); thymol (Lot 171210); magnolol (Lot 171126); amygdalin (Lot 170902); paeoniflorin (Lot 180124) were purchased from Beijing Shengshi Kangpu Chemical Engineering Technology Institute, Ephedrine Hydrochloride (Lot: 171241–201508) was purchased from China Research Institute of Food and Drug Verification. 500 mg of fine particles of each TCM was dissolved in 25 mL of 50% methanol solution, ultrasonic cleaning for 30 min, and filtered with 0.45 μm microporous filters. High performance liquid chromatography (HPLC) was performed to identify the main chemical constituents in the TCMs. Fingerprints of TCMs were read, and some chemical constituents of TCMs were identified according to the spectrograms and retention times of their standards (Fig. 1).

**Table 1:** The compositions of Traditional Chinese Medicine

Traditional Chinese Medicine	No.	Herbal drug	Official name	Local name	Batch number	Collection place
Yinqiao powder	1	Fructus Forsythiae	*Forsythia suspensa* (Thunb.) Vahl	Lianqiao	1712002S	Shanxi
	2	Flos Lonicerae	*Lonicera japonica* Thunb.	Jinyinhua	1801002S	Shandong
	3	Radix Platycodonis	*Platycodon grandiflorus* (Jacq.) A.DC.	Jugeng	1712004S	Anhui
	4	Herba Menthae	*Mentha haplocalyx* Briq.	Bohe	1709001S	Jiangsu
	5	Herba Lophatheri	*Lophatherum gracile* Brongn.	Danzhuye	1712002S	Sichuan
	6	Radix Glycyrrhizae	*Glycyrrhiza uralensis* Fisch.	Gancao	1801016S	Gansu
	7	Herba Schizonepetae	*Schizonepeta tenuifolia* Briq	Jingjiesui	1710001S	Hebei
	8	Fermented soybean	*Glycine max* (L.) Merr.	Dandouchi	1704001S	Henan
	9	Fructus arctii	*Arctium lappa* L.	Niubangzi	1801001S	Gansu
	10	Rhizoma Phragmitis	*Phragmites communis* Trin.	Lugen	1709002S	Anhui
Xinjiaxiangruyin	1	Herba Moslae	*Mosla chinensis* Maxim.	Xiangru	1708002S	Jiangxi
	2	Flos Lonicerae	*Lonicera japonica* Thunb.	Jinyinhua	1801002S	Shandong
	3	Dolichos	*Dolichos aciphyllus* R.Wilczek	Biandouhua	161001	Guangdong
	4	Cortex Magnoliae Officinalis	*Magnolia officinalis* Rehder & E.H.Wilson	Houpu	1712003S	Sichuan
	5	Fructus Forsythiae	*Forsythia suspensa* (Thunb.) Vahl	Lianqiao	1712002S	Shanxi
Guizhi-and-Mahuang decoction	1	Herba Ephedrae	*Ephedra intermedia* Schrenk & C.A.Mey.	Mahuang	1706004S	Neimenggu
	2	Ramulus Cinnamomi	*Cinnamomum cassia* (L.) J.Presl	Guizhi	1801003S	Guangxini
	3	Semen Armeniacae Amarum	*Prunus armeniaca* L.	Xingren	1712003S	Hebei
	4	Radix glycyrrhizae preparata	*Glycyrrhiza uralensis* Fisch.	Zhigancao	1712003S	Gansu
	5	Radix Paeoniae Alba	*Paeonia lactiflora* Pall.	Shaoyao	1801001S	Anhui
	6	Radix Glycyrrhizae	*Glycyrrhiza uralensis* Fisch.	Gancao	1801016S	Gansu
	7	Rhizoma Zingiberis Recens	*Zingiber officinale* Roscoe	Shengjiang	–	Guangdong
	8	Jujube	*Ziziphus jujuba* Mill.	Dazao	–	Guangdong

### Animals

C57BL/6 wild type (WT) mice of SPF grade were purchased from Medical Animal Experiment Center of Guangdong Province (animal license #: SCXK 2013-0002). TLR7 knockout (TLR7 KO) mice were provided by the Jackson Laboratory (USA) and all the knockout mice were returned to C57BL/6 mice over ten generations. The breeding and feeding of TLR7 KO mice were carried out in SPF environment of Animal Experimental Center of Jinan University with free drinking water and feeding, in temperature-controlled animal facility (temperature: 20 ± 2 °C; humidity: 50%) with 12 h diurnal cycle and individual ventilated cages (IVC). Sixty WT female C57BL/6 mice (6–8 weeks old) were randomly divided into six experimental groups (n = 10/group): blank control group, virus control group, oseltamivir group (positive control group), Xinjiaxiangruyin group, Guizhi-and-Mahuang decoction group, and Yinqiao powder group. The same protocol was applied to TLR7 KO mice. Mice were anesthetized by intraperitoneal injection of 130–160 μL 6% chloral hydrate solution, and 50 μL sterile solution of 0.9% NaCl was dropped nasally in blank control group. The rest groups were challenged with 50 μL influenza virus FM1 suspension (dilution 1:640) nasally. None of the mice infected died. The drug dose was calculated based on body-weight differences between humans and mice and the dose for the mice was equivalent to 9.1 times that for human clinical dosage. 24 h after infection, 0.4 mL of Yinqiao powder (560 mg/mL), Xinjiaxiangruyin (230 mg/mL), Guizhi-and-Mahuang decoction (350 mg/mL) and oseltamivir (1.5 mg/mL) were given to mice in treatment groups by gastric gavage once a day for 5 days, respectively. The blank control group and virus control group were treated with the same amount of double distilled water. The mice were placed in an artificial climate incubator (temperature: 18–20 °C; humidity: 50%; light: 3000Lx) and a free diet during the experiment and were observed for 5 days. The mice were sacrificed to collect lung and spleen for examination 6 days after infection.

### Virus strains

Virus strain: type A influenza virus, FM1 mouse lung adapted strain (FM1), stored at − 80 °C. It was provided by the Department of Microbiology and Immunology, School of Basic Medical Sciences, Jinan University. The hemagglutination titer was 1:40 after two times of routine chick embryo resuscitation. The mortality of mice 14 days after virus infection at different concentrations was determined by double dilution method. The virus concentration causing 20% mouse mortality (blood coagulation titer 1:640) was used and 50 μL of virus solution was given to each mouse.

### The changes of body weight, animal condition, survival rate and lung index

Starting at 2 days before infection, the weight of each mouse was recorded at the same time point every day. The changes of symptoms, water and food intake, hair color, activity, survival time, death condition and so on were observed twice a day. The mice were sacrificed 6 days after infection for lung tissue collection. Adipose tissue was removed, and the lung tissue was washed with sterile phosphate buffer saline (PBS). The lungs were then dried with filter paper and weighed. The lung index was calculated using the formula: lung index = lung weight/body weight × 100%.

### Observation of pathological changes in lung tissue

The fresh lungs were fixed in 4% paraformaldehyde, dehydrated, embedded in paraffin wax and serially sectioned at 5 µm. Haematoxylin and eosin (H&E) was employed and the pathological changes of the lung tissue were observed under light microscope.

### Detection of the level of Th17 and Treg cells in the spleen of mice

The spleens of mice were rinced with RPMI-1640 medium and continuously grounded. The resultant cell suspension was placed on the upper layer of the lymphocyte separating fluid (Multi Sciences, China). After centrifugation, the intermediate white lymphocyte layer was collected and washed with PBS. Lymphocytes were then resuspended in RPMI-1640 medium containing 10% fetal bovine serum (FBS) and adjusted to 1 × 10^6^/mL concentration. To detect Treg cells, 100 μL of cell suspension was incubated with anti-CD4 and anti-CD25 antibodies for 30 min at 4 °C avoiding light, and then washed with precooled PBS. Cells were then resuspended in film breaking working fluid (fixation/permeabilization concentrate: fixation/permeabilization diluent = 1:3) (eBioscience, USA), and incubated for 30 min at 4 °C avoiding light. After washed with 1 × Permeabilization buffer (eBioscience, USA), intracellular antibody Foxp3 was added and incubated for 30 min at 4 °C avoiding light. Finally, cells were washed and resuspended in 200 μL of PBS for analysis on FACSVerse Flow cytometry (Becton–Dickinson Bioscie NCS, Franklin Lakes, NJ, USA). Flowjo7.6.1 (Flow Jo, Ashland, OR, USA) software was used to process and analyze the experimental data. The Antibodies were shown in Table 2.

**Table 2:** Antibodies used for flow cytometry

Antibody	Clone	Company	Labels used
CD4	RM4-5	eBioscience	PE
IL-17A	eBio17B7	eBioscience	FTIC
CD25	PC61.5	eBioscience	APC
Foxp3	FJK-16 s	eBioscience	PE-cy5.5

*PE* phyco-erythrin, *FITC* Fluorescein isothiocyanate, *APC* allophycocyanin, *CD4* cluster of differentiation 4, *IL-17A* interleukin-17A, *CD25* cluster of differentiation 25, *Foxp3* forkhead box P3

### Detection of mRNA expression levels of TLR7, MyD88, IRAK4 and NF-κB in mouse lung with RT-qPCR

The lung tissue was collected for total RNA extraction using Trizol (TaKaRa, Japan) according to the manufacturer’s instructions. First-strand cDNA synthesis and the SYBR^®^ Green qPCR assay were performed using the PrimeScript™ RT Reagent Kit (TakaRa, Japan). The reverse transcription reactions were performed in the Bio-Rad S1000™ thermocycler (Bio-Rad, USA). qPCR protocol was: 95 °C, 30 s; 95 °C, 5 s; 60 °C, 30 s; with 40 amplification cycle; 95 °C, 10 s using the ABI 7000 Real Time PCR machines. All primers (Table 3) were designed and synthesized by Shanghai Generay Biotech Co. Ltd. Corresponding relative mRNA expression was calculated by the 2^−ΔΔCt^ method [37]. mRNA expression levels of FM1, TLR7, MyD88, IRAK4 and NF-κB were evaluated using GAPDH as an internal standard control.

**Table 3:** Primers used for RT-qPCR studies

Gene	Forward (5′ to 3′)	Reverse (5′ to 3′)
FM1	GACCAATCCTGTCACCTCTGAC	AGGGCATTNTGGACAAAGCGTCTA
GAPDH	CTGAGCAAGAGAGGCCCTATCC	CTCCCTAGGCCCCTCCTGTT
TLR7	GGGTCCAAAGCCAATGTG	TGTTAGATTCTCCTTCGTGATG
MyD88	CGATTATCTACAGAGCAAGGAATG	ATAGTGATGAACCGCAGGATAC
IRAK4	CATCGTGGCGGTGAAGAAG	AGCATACACTAAGCACAGGTTG
NF-κB	ATTCTGACCTTGCCTATCTAC	TCCAGTCTCCGAGTGAAG

*GAPDH* glyceraldehyde-3-phosphate dehydrogenase, *TLR7* Toll-like receptor 7, *MyD88* Myeloid differentiation primary response 88, *IRAK4* interleukin-1 receptor-associated kinase 4, *NF-κB* nuclear factor kappa-light-chain-enhancer of activated B cells

### Determination of protein expression levels of TLR7, MyD88, IRAK4 and NF-κB by Western-blotting

Protein samples were extracted from lung tissue homogenate using a RIPA lysis buffer (Multi Sciences, China) supplemented with protease and phosphatase inhibitors, and the protein concentrations were quantified using BCA assay. Separated with 10% SDS-PAGE, the protein was transferred wetly to the PVDF membrane (Millipore, USA). The PVDF membrane was blocked in TBST containing 5% skimmed milk and incubated with the Rabbit monoclonal antibody (mAb) (CST, USA) of GAPDH, TLR7, MyD88, IRAK4 and NF-κB overnight at 4 °C, respectively, and then incubated for 2 h in the HRP-labeled secondary antibodies against rabbit (Multi Sciences, China). The blots were developed using the ECL color display kit (Multi Sciences, China), and ImageJ image analysis software was used for ALIANCE gel image analyzer and imaging.

### Statistical analysis

The experiment data were processed and analyzed with the statistical software SPSS 13.0. All results were presented as mean ± standard deviation (x ± s). Two groups of independent samples were compared by t-test. Multiple experimental groups were analyzed by ANOVA in advance, and SNK was used for comparison of each two groups according to the homogeneity of variance test. P < 0.05 indicated that the difference was statistically significant, and P < 0.01 means significant difference.

## Results

### Guizhi-and-Mahuang decoction had a protective effect on virus infection

**Fig. 2:**
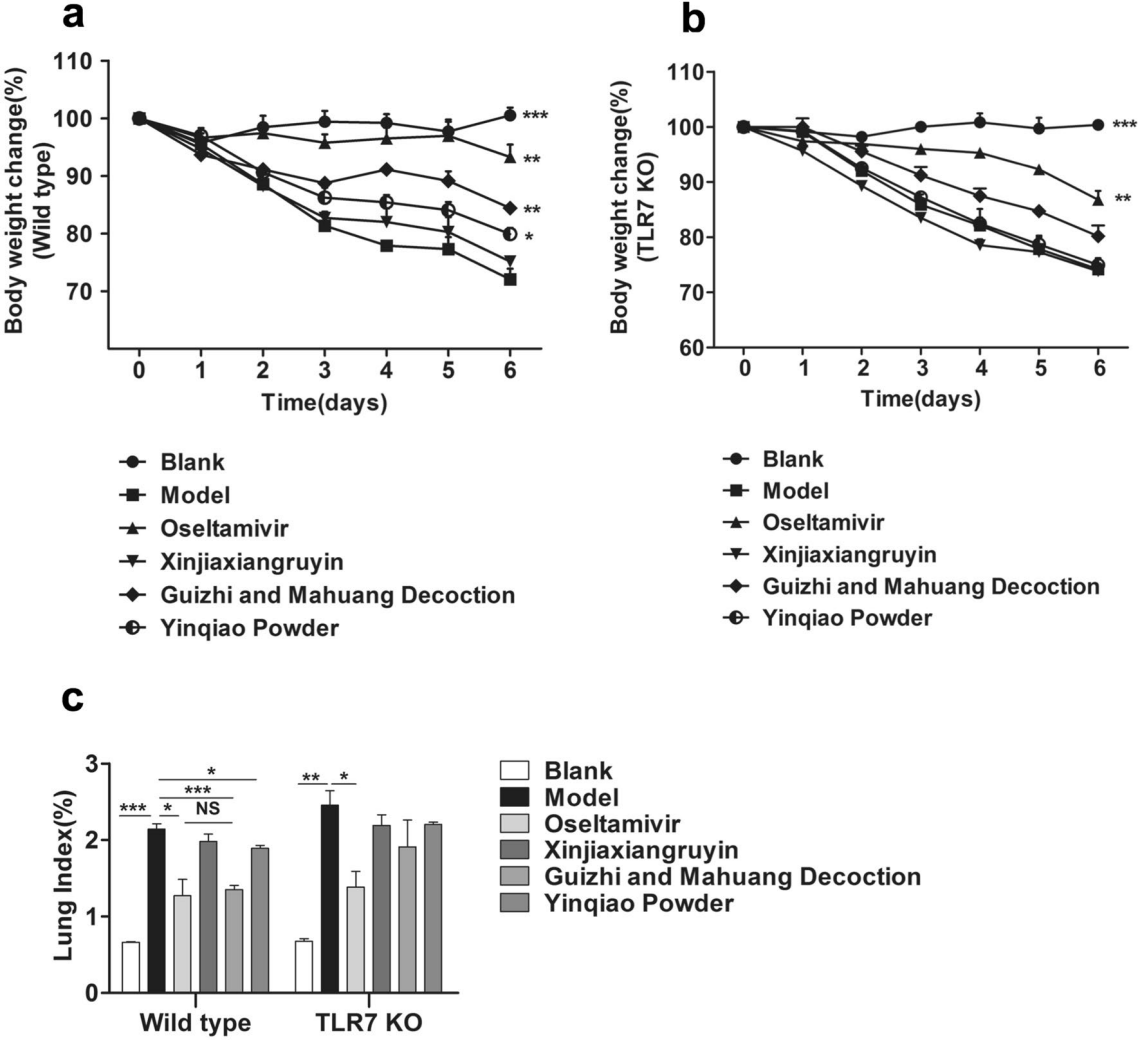
Effects of three TCM compounds on body weight loss and lung index of mice infected with influenza virus. C57BL/6 wild type (**a**) and TLR7 KO (**b**) mice were infected with 50 μL FM1 virus and then were gavaged with distilled water, containing Oseltamivir, Xinjiaxiangruyin, Guizhi-and-Mahuang decoction or Yinqiao powder daily 24 h after virus infection, respectively. Changes in body weight of each mouse were recorded. Each group was compared to the model group. **c** The body weight of infected mice and blank were monitored every day for 6 days. The mice were sacrifice on day 6, and the total lung were completely removed and weighed. Lung index = lung weight/body weight × 100%. *P < 0.05, **P < 0.01, ***P < 0.001

To evaluate the effects of TCMs on anti-virus and development of lung inflammation, we infected wild-type and TLR7 KO C57BL/6 mice with Influenza virus FM1 and applied Oseltamivir and three TCMs 24 h post-infection for 5 days, respectively. In wild-type mice, the animals in sham group had good mental state, good hair color, quick action, breathing, normal gait and natural weight growth. Clinical symptoms in virus infected mice were apparent 2 days after infection. In viral control group, mice had typical flu symptoms, including hair discoloring, towering hair, curled up, arch, paralysis, loss of appetite, reduced water drinking, convulsions, faint and breathing difficulties. Meanwhile the body weight was decreased gradually. Compared to virus control, animals in Oseltamivir group, Guizhi-and-Mahuang decoction group were significantly improved with towering hair, convulsions and other symptoms remarkably reduced. Yinqiao powder group only showed slightly improvement. The body weight changes in each group were shown in Fig. 2a. The average body weight of mice on day 6 in virus control group, Xinjiaxiangruyin group and Yinqiao powder group were decreased to 72.08%, 75.16% (NS) and 79.94% (P < 0.05) of the original body weight, respectively, while mice in Oseltamivir group and Guizhi-and-Mahuang decoction group were better with 93.29% (P < 0.01) and 84.45% (P < 0.01) of the original body weight, respectively. In TLR7 KO mice, we observed that, except for the blank group and the Oseltamivir group, the body weight of the virus control group and the three TCM groups decreased significantly (Fig. 2b). We further weighed the whole lung of each mouse and calculated the lung index. As shown in Fig. 2c, in both wild type and TLR7 KO mice, there was a significant difference in the lung index between the blank group and the virus control group (P < 0.01), indicating that the viral infection was successful. As a positive control drug, Oseltamivir significantly reduced lung index compared to the virus control group (P < 0.05). In wild type mice, the lung indexes of animals in Guizhi-and-Mahuang decoction group and Yinqiao powder were significantly decreased compared to the virus control group (P < 0.05); and there was no significant difference (NS) between Guizhi-and-Mahuang decoction group and Oseltamivir group. In TLR7 KO mice, the three TCM groups had no significant difference compared to the virus control group or among three groups (NS).

### Pathological changes of lung tissue

**Fig. 3:**
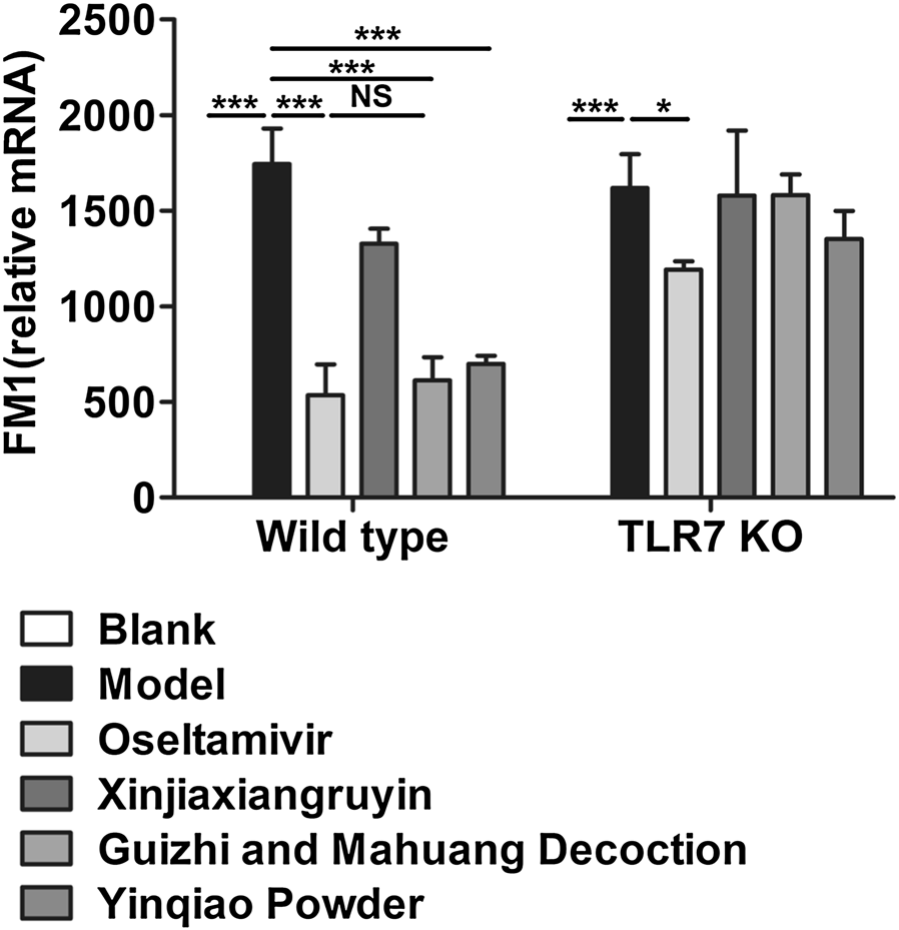
The influenza FM1 viral load in the lung tissue of wild type and TLR7 KO mice infected with influenza virus on day 6. *P < 0.05, **P < 0.01, ***P < 0.001

The amount of replicating virus in the lungs is thought to correlate with the degree of lung pathological changes. Therefore, wild type and TLR7 KO mice were infected with Influenza FM1 virus, and four drugs were used to treat the mice 24 h after infection, respectively. After 5 days of continuous gavage, lungs of the mice were collected. The RNA was extracted from the left lung, and the replication of virus in each group was evaluated. We observed that the virus control group had a significantly higher viral mRNA expression level in lung tissue than blank group in wild type mice (P < 0.001), Meanwhile, Oseltamivir, Guizhi-and-Mahuang decoction and Yinqiao powder dramatically decreased the viral load compared to the virus control group (P < 0.001). In TLR7 KO mice, the viral load of virus control group was significantly higher than that of blank control group (P < 0.001), but only Oseltamivir group reduced the replication of virus (P < 0.05). We then performed comparative analysis between wild type and TLR7 KO mice, and found no difference between virus control groups. However, Oseltamivir, Guizhi-and-Mahuang decoction and Yinqiao powder treatment induced significant difference between wild type and TLR7 KO mice, suggesting that TLR7 receptor played a key role in antivirus effects (Fig. 3).

**Fig. 4:**
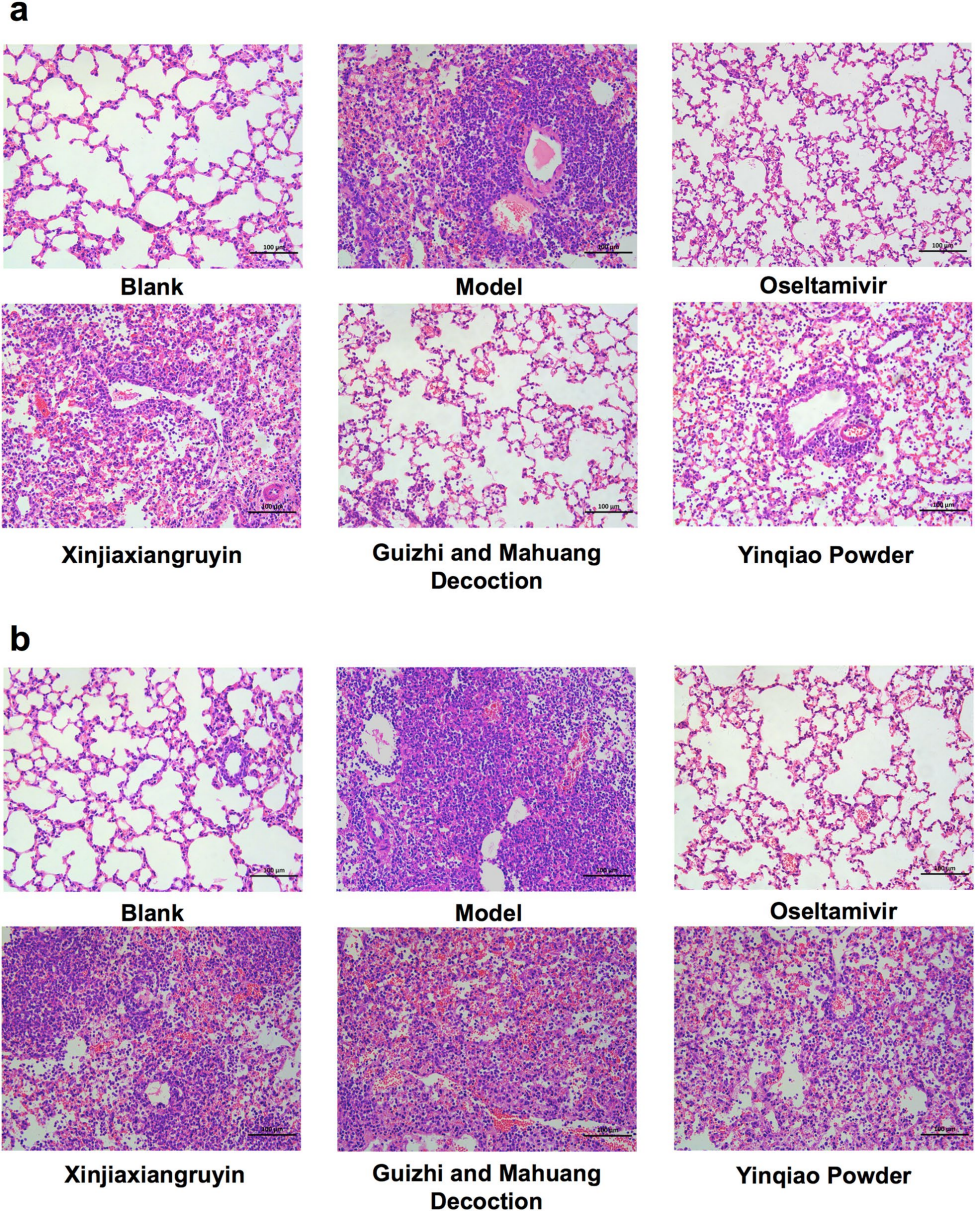
Lung histopathology in wild type and TLR7 KO mice infected with influenza FM1. The lungs were collected on day 6 postinfection, and sections were prepared for histopathological analysis. **a**, **b** Indicate C57BL/6 wild mice and TLR7 KO mice, respectively. This figure shows representative results of experiments with ten mice in each group. Bars = 100 μm

At the same time, the right lungs of mice were fixed in 4% formaldehyde solution and HE staining was performed. In wild type mice (Fig. 4a), blank control group showed clear and intact alveolar structure. The alveolar wall was thin, and there was no inflammatory secretion in alveolar cavity, nor alveolar interstitial infiltration of inflammatory cells. In virus control group, inflammatory damage was observed in lung tissue, including a large number of inflammatory cell infiltration in alveolar cavity, alveolar septum thickening and severe interstitial edema, vascular congestion, bronchial obstruction due to inflammatory exudation. Formation of cavitation was also observed after epithelial cell shedding or necrosis. In the positive control group (oseltamivir group), alveolar inflammatory cells were significantly reduced and peribronchial alveolar wall was thinning with no inflammatory cells, similar to that in the blank control group, and only alveolar wall thickening was observed. In Xinjiaxiangruyin group, alveolar structure destruction was increased. Bronchial wall and alveolar septa were thickened, and there were a large number of infiltrating mononuclear cells, with bronchiole having more infiltrating inflammatory cells. In Guizhi-and-Mahuang decoction group, the inflammatory lesion was more significantly reduced than that in virus control group. The alveolar wall was thin, with less infiltration of mononuclear cells, capillary dilatation and congestion of alveolar wall. The alveolar septum was not obviously thickened, with only a small number of mononuclear cells and lymphocytes. There was no inflammatory exudation in bronchiole, similar to that in the positive control group. In Yinqiao powder group, the alveolar wall was thickened, but alveolar infiltration was not obvious. Compared to the virus control group, the alveolar infiltration and inflammatory reaction was milder and there was no exudate in the bronchiole. In general, the influenza viral infection caused acute inflammatory responses and mononuclear cell infiltration in the respiratory tissue, which contributed to the main pathological basis of the disease. In TLR7 KO mice (Fig. 4b), the Oseltamivir treatment remarkably reduced inflammation compared to virus control. However, neither TCM treatment groups showed any difference compared to the virus control, which indicated that the therapeutic effects of TCM on viral infection induced inflammation needed the participation of TLR7 signaling.

### Profiling of Th17 cells and Treg cells in splenocytes

**Fig. 5:**
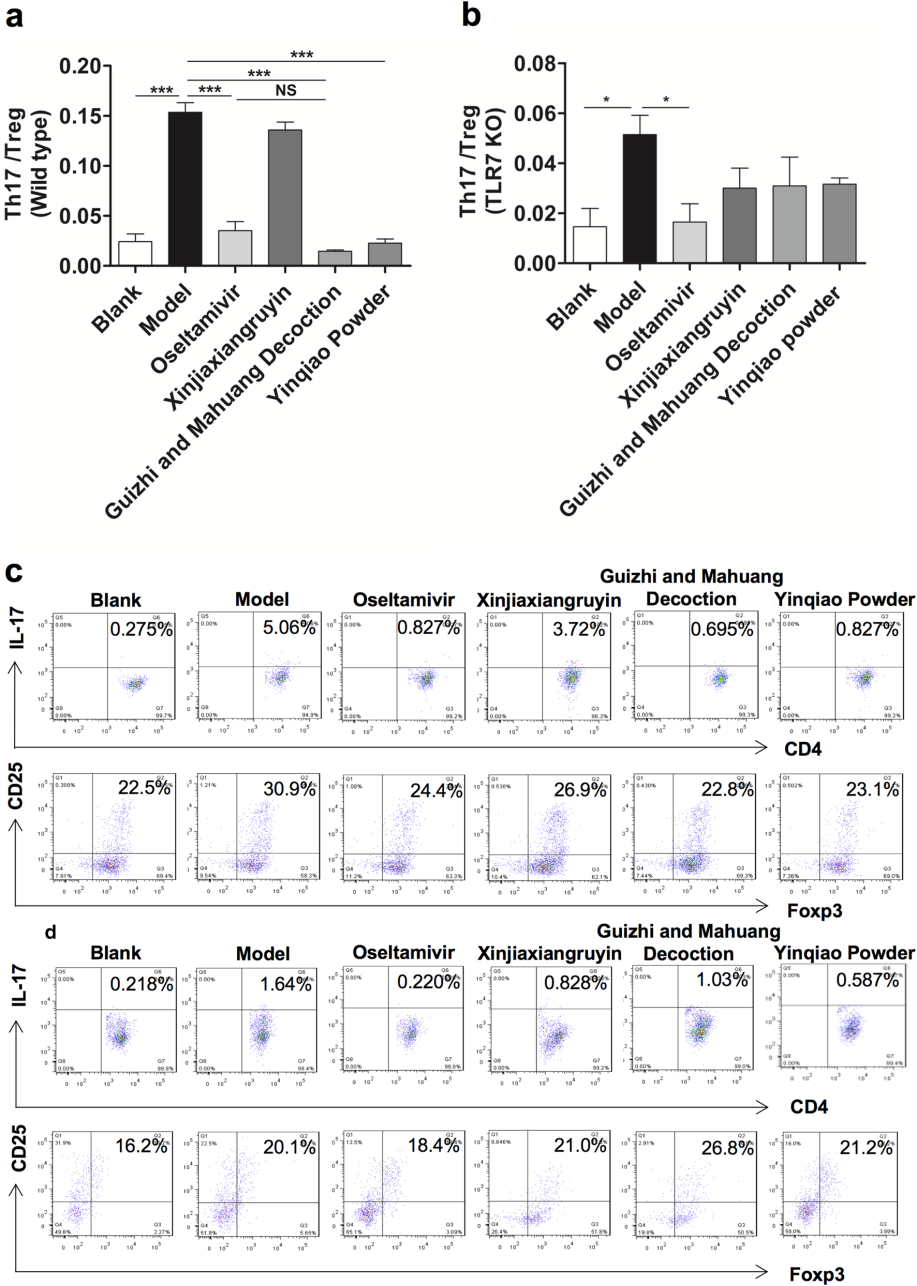
Profiling of Th17 cells and Treg cells in splenocytes of mice. C57/6 wild type mice (n = 10/group) and TLR7 KO mice (n = 10/group) were infected with 50 μL of influenza FM1 virus. Splenocytes were isolated from 3 mice out of each group on day 6 post-infection and Th17 cell and Treg cell response were assayed in wild type mice (**a**) and TLR7 KO mice (**b**). Data are presented as the mean ± SEM. Th17 cell response was determined by intracellular CD4, IL-17 staining. Treg cell response was determined by intracellular CD4, CD25 and Foxp3 staining. Data are presented as representative density plots in wild type mice (**c**) and TLR7 KO mice (**d**). *P < 0.05, **P < 0.01, ***P < 0.001

In the process of immuno-regulation, Th17 cells are closely related to Treg cells. IL-17 secreted by Th17 cells can aggravate the inflammatory responses, while Treg cells can reduce the production of inflammatory cytokines and antibody secretion to inhibit immune responses. Th17/Treg balance plays an important role in maintaining the immune homeostasis of the body. In order to further verify the inflammatory response in mice, we used flow cytometry to detect the splenocytes in mice. As shown Fig. 5, in the wild type mice, the ratio of Th17 cells and Treg cells in the virus control group was significantly higher than that in the blank control group (P < 0.001). Compared to the virus control, except for Xinjiaxiangruyin group, ratios of Th17 cells and Treg cells in other three groups were significantly decreased (Fig. 5a). In TLR7 KO mice, the ratio of Th17 cells and Treg cells in the virus control group was higher than that in the blank control group. Compared to the virus control group, only the Oseltamivir treatment resulted in significant decrease in the Th17 cell and Treg cell ratio (P < 0.05) (Fig. 5b).

### Effects of drugs on TLR7/NF-κB signaling pathway

**Fig. 6:**
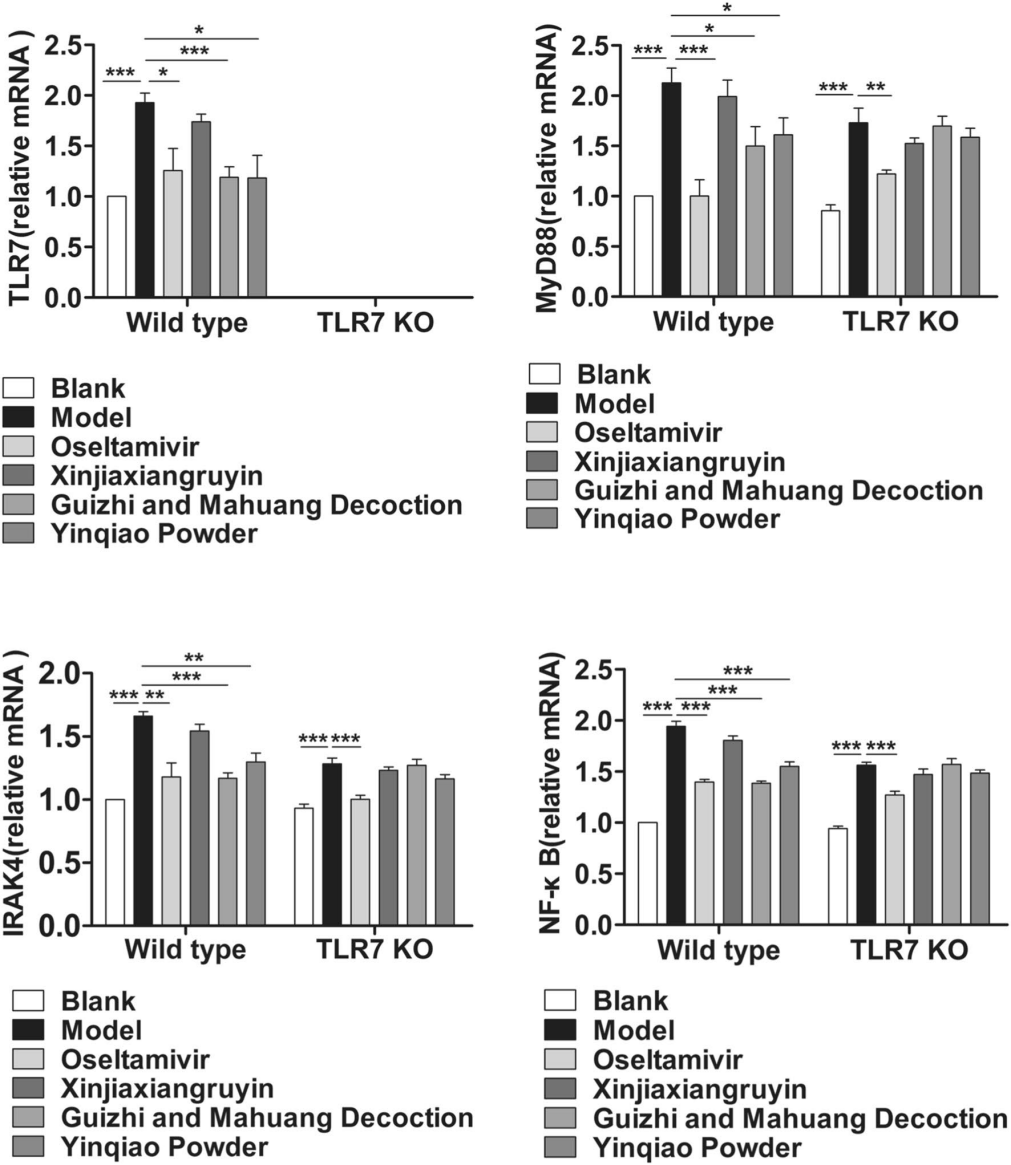
The effect of different drugs on the mRNA expression of TLR7 and NF-κB signaling pathway in lung tissue of influenza virus infected mice. *P < 0.05, **P < 0.01, ***P < 0.001

**Fig. 7:**
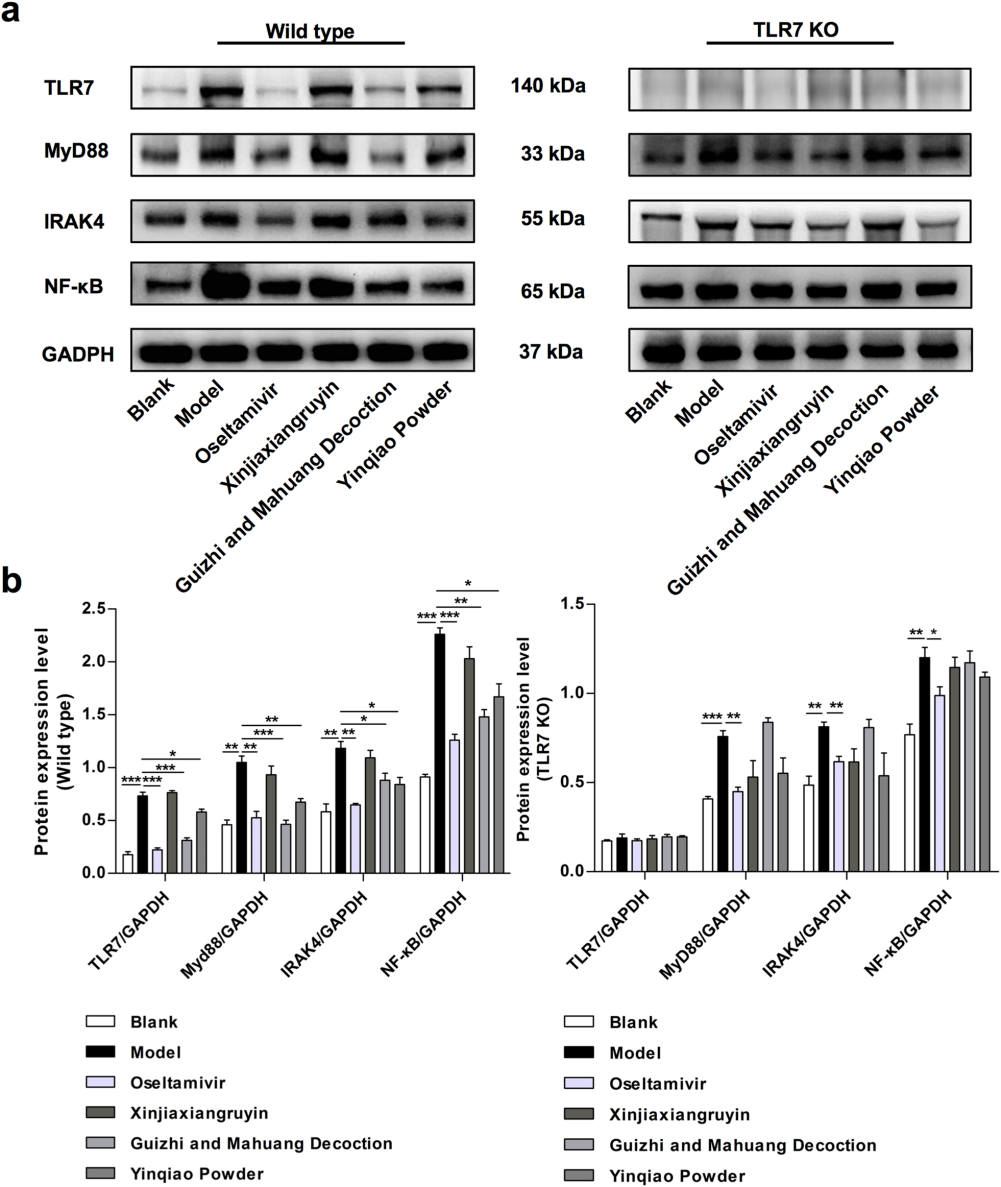
The effect of different drugs on the protein expression of TLR7 and NF-κB signaling pathway in lung tissue of influenza virus infected mice. **a** Proteins were evaluated by western blotting assay. **b** Quantification of TLR7, MyD88, IRAK4, NF-κB protein was detected by densitometric analysis. *P < 0.05, **P < 0.01, ***P < 0.001

In order to further explore the contribution of Chinese herbal compounds to innate immune response after influenza virus infection, wild type and TLR7 KO mice were infected with influenza virus, respectively. 6 days after infection, the mRNA relative expression and protein levels of components in TLR7 and NF-κB signaling pathways in lung tissue were detected with RT-qPCR and western blotting, respectively. As shown in Fig. 6, virus infection induced significant increase in the mRNA levels of TLR7, MyD88, IRAK4 and NF-κB in wild type mice compared to blank control (P < 0.001). Compared to the virus control group, all treatment, except for Xinjiaxiangzhuyin, resulted in significant decrease in expression levels of TLR7, MyD88, IRAK4 and NF-κB (P < 0.05), with Oseltamivir and Guizhi-and-Mahuang decoction treatment having more reduction, followed by Yinqiao powder. These data indicated that influenza virus infection could activate TLR7/NF-κB signaling pathway, while Oseltamivir and Guizhi-and-Mahuang decoction significantly suppressed their expression. We also tested the TLR7 KO mice at the same time. The relative mRNA expression of TLR7 was significantly lower than that in wild type mice, and had no difference among all experimental groups (NS), which confirmed the success of TLR7 KO. Then, we studied the effect of TLR7 gene silencing on the downstream signaling pathway, and found that MyD88 and NF-κB were still activated. We further demonstrated that virus infection increased protein expression levels of TLR7, MyD88, IRAK4 and NF-κB with western blotting, while Oseltamivir, Guizhi-and-Mahuang decoction, Yinqiao powder decreased their expression. The protein levels in each group were in accordance with the expression of mRNA (Fig. 7).

## Discussion

Viruses activate immune pathways through three pattern recognition receptors (PRRs), TLRs, RLHs and NLRs (nucleotide-oligomerization domin (NOD) -like receptors) [38–40]. TLR7 recognizes the single-stranded RNA viruses, such as vesicular stomatitis virus and influenza virus [29]. Some studies have shown that TLR7 is mainly expressed in lung, placenta, heart, spleen, bone marrow, lymph nodes and other tissues [34, 41], so we detected mRNA and protein expression levels of TLR7 and its related genes in lung tissue in a mouse model. We infected wild type and TLR7 KO C57BL/6 mice with influenza virus in a normal environment, and applied various TCM treatment to evaluate their anti-virus and anti-inflammation effects. The viral load of the lung tissue in the virus control group was significantly higher than that in the blank control group, indicating that the experimental animal model was successfully established.

The drugs used in our study was selected according to the three main dialectic methods of TCM at the onset of diseases: acrid-warm herbs relieving superficies (Yinqiao powder), cold-pungent diaphoresis (Guizhi-and-Mahuang decoction), and clearing damp (Xinjiaxiangruyin). In different environments, drugs may perform differently against influenza, and our experiment was carried out in the normal environment (temperature: 18–20 °C, humidity 50%, and light: 3000Lx) for the viral infection and drug administration in mice. The experimental results indicated that under normal circumstances, Guizhi-and-Mahuang decoction and Yinqiao powder had defensive effects against influenza virus infection, while the effect of Xinjiaxiangruyin was not obvious in wild type mice. Compared to the positive control oseltamivir, Guizhi-and-Mahuang decoction showed no difference in the lung index, lung pathology change, virus load and the ratio of Th17 cells and Treg cells, demonstrating its efficacy in anti-virus and the potential as a new anti-influenza drug. Xinjiaxiangruyin may play a good role of anti-influenza in hot and humid environment, which warrants further research and exploration.

In terms of inflammatory response, we further demonstrated the role of Chinese herbal compounds in the anti-influenza process. Th17 cells secrete IL-17 as well as cytokines such as IL-21 and IL-22, and IL-17 can aggravate inflammatory reaction and participate in various autoimmune diseases [42]. Treg is a CD4+ T cell subset with immunosuppressive activity. Treg cells release cytokines IL-10 and TGF- beta to inhibit the function of T cells and antigen presenting cells, and reduce the production of inflammatory cytokines and antibody secretion. Foxp3 is an important transcription factor of Treg, and its continuous expression is the key factor in maintaining the inhibitory activity of Treg. Foxp3+ Treg cells have the function of anti-inflammatory and maintenance of autoimmune tolerance [43]. Foxp3+ Treg cells and Th17 cells inhibit each other in differentiation, development and function [44]. Therefore, Foxp3+ Treg/Th17 balance plays an important role in maintaining immune homeostasis.

Mononuclear cells were isolated from spleen of mice and flow cytometry was used to detect and analyze Th17 cells and Treg cells. The ratio of Th17 to Treg in the virus control group was much greater than that in the blank control group. In wild type mice, Oseltamivir, Guizhi-and-Mahuang decoction, and Yinqiao powder could decrease the ratio of Th17/Treg, which indicated that Treg cells inhibited the release of inflammatory factors and alleviated the inflammatory response in the lungs of mice. In TLR7 KO mice, the deletion of TLR7 gene significantly decreased the antiviral effect of Guizhi-and-Mahuang decoction and Yinqiao powder, which indicated that these two drugs might function through TLR7. The inflammatory response in lung pathology was consistent with the ratio change of Th17/Treg cells.

At both mRNA and protein expression levels, we have demonstrated the effect of TCMs on the TLR7/NF-κB signaling pathway in the process of anti-influenza virus infection. It’s found that the TLR7 was up-regulated in the macrophages upon virus infection [45]. Studies have shown that TCMs can play a role in the treatment of influenza by TLR7 mediated MyD88-dependent signaling pathway. In this study, we mainly examined the mRNA and protein expressions of components in TLR7-mediated MyD88-dependent signaling pathway. The results showed that the expression levels of TLR7, MYD88, IRAK4 and NF-κB were remarkably up-regulated upon viral infection, and Guizhi-and-Mahuang decoction and Yinqiao powder could down-regulate their expressions in wild type mice. In TLR7 KO mice, the mRNA and protein expressions of TLR7 were very low in all groups, indicating that the TLR7 gene was successfully knocked out. However, MyD88 was up-regulated upon viral infection, because all other members of TLRs family, except for TLR3, can be activated by MyD88 dependence [46]. The expression of IRAK4 was low upon viral infection, indicating that the TLR7 knockout might affect IRAK4 expression in the downstream pathway. TLR7 knockout decreased the transcription and translation levels of NF-κB overall, which suggested that TLR7 plays an indispensable role in the pathway. However, there was no significant change in survival rate of TLR7 KO mice, which was probably due to the compensatory role of the RLH signaling pathway. RIG-I (RIG-like receptors) is also a type of pattern recognition receptor [47] and involves in the recognition of influenza virus [48]. NF-κB is the common nuclear factor of TLR7 and RIG-I, so when TLR7 gene is knocked out, RIG-I may play a compensatory role, and ultimately activate NF-κB to maintain stable expression.

Oseltamivir is a neuraminidase inhibitor [49], which blocks the activity of neuraminidase, inhibiting the replication and toxicity of virus so as to effectively prevent and even alleviate the symptoms of influenza. It can be combined with the flu vaccine without affecting the antibody production. However, oseltamivir resistant influenza A and influenza B virus strains have continuously emerged in recent years [5], and many side effects have been reported, such as vomiting, nausea, headache, and rash [50]. TCM has a lot of advantages. It not only has the functions of inhibiting virus replication, preventing cytopathic effect, improving host immunity and blood circulation, but also greatly reduces the drug resistance due to its compatibility, which makes TCM a unique and effective option in the prevention and treatment of influenza.

## Conclusion

The results of this study show that, under normal circumstances, Guizhi-and-Mahuang decoction and Yinqiao powder play a more significant role in the process of anti-influenza virus. After influenza virus infection, transcription and translation levels of components in the TLR7/NF-κB pathway were increased in mouse lungs, and Guizhi-and-Mahuang decoction and Yinqiao powder effectively reduced those changes to achieve viral clearance and inflammation recovery. Although different from oseltamivir in antiviral mechanisms, Guizhi-and-Mahuang decoction showed similar effects on therapeutic effects.

## Additional file

**Additional file 1.** Minimum Standards of Reporting Checklist.
